# Incidence and predictors of mortality among low birth weight neonates admitted to neonatal intensive care unit in selected public hospitals, North Shoa zone, Amhara Region, Ethiopia: multi-center retrospective follow-up study

**DOI:** 10.3389/fped.2025.1524966

**Published:** 2025-06-11

**Authors:** Leweyehu Alemaw Mengstie, Mohammed Tessema Gebeyehu, Wegayehu Zeneb Teklehaimanot, Wondimeneh Shibabaw Shiferaw, Tirusew Nigussie Kebede, Worku Abemie, Bekahegn Girma

**Affiliations:** ^1^ School of Nursing and Midwifery, Asrat Woldeyes Health Sciences Campus, Debre Berhan University, Debre Berhan, Ethiopia; ^2^ University of Queensland Centre for Clinical Research, Faculty of Medicine, The University of Queensland, Herston, QLD, Australia

**Keywords:** low birth weight, incidence, survival status, predictors, Ethiopia

## Abstract

**Background:**

Birth weight of less than 2,500 g is the leading cause of neonatal morbidity and mortality. Various studies in developed and developing countries examine the prevalence and associated factors. However, little is known about low birth weight neonatal incidence and predictors of Mortality in Ethiopia.

**Objective:**

The study aimed to assess the incidence and predictors of mortality among low birth weight neonates admitted to the neonatal intensive care unit at a public hospital, in Northeast, Ethiopia, 2021.

**Methods:**

An institution-based retrospective follow-up study was conducted among 416 low-birth-weight neonates admitted to the neonatal intensive care unit from January 1, 2019 to December 30, 2021. Epi-data version 3.1 was used for data entry and Stata14 software for analysis. The Kaplan–Meier survival curve and the Log-rank test were used to estimate the cumulative survival time and compare the probability of survival time among variables. Multi-variable and bi-variable Cox proportional hazard model was used to identify predictor variables.

**Results:**

Out of 416 Low birth weight neonates, 107 (25.72%) (95% CI: 21.51, 29.93) of them died. The overall incidence rate of mortality was 42.83 per 1,000 (95% CI: 35.34, 51.77) with 2,498 person-days of observation. Twin pregnancy [adjusted hazard ratio (AHR): 1.7 (95% CI: 1.42, 3.29)], Sepsis (AHR: 1.5; 95% CI: 1.01, 2.32), respiratory distress syndrome (AHR: 1.6; 95% CI: 1.15, 2.68), maternal history of HIV (AHR: 2.2; 95% CI: 1.54, 3.42), maternal DM (AHR: 2.5; 95% CI: 1.70, 3.87) and preeclampsia (AHR: 1.8; 95% CI: 1.23, 2.73) were found to be significant predictors of low birth weight (LBW) neonatal mortality.

**Conclusion:**

The incidence rate of low birth weight neonatal mortality was high and continues as a public health issue. Therefore, special attention should be given to those identified predictors of mortality, and different measures should be implemented to prevent premature birth in the high-risk population by improving prenatal care.

## Introduction

A low birth weight (LBW) neonate is a baby whose first measured weight is less than 2,500 g without gestational age consideration (1). It is one of the significant determinants of perinatal survival, infant morbidity, mortality, and the risk of developmental disabilities and illnesses in future lives (2).

There were more than 20.5 million LBW neonates in 2015, and more than 96% of LBW newborns were born in developing countries (3). Globally, the incidence and mortality of LBW infants are still high and considered a significant cause of neonatal mortality, especially in developing countries. It contributes to 60%–80% of all neonatal deaths annually (4).

Almost all neonatal deaths (98%) occurred in low- and middle-income countries (LMICs), with 75% occurring in Southern Asia and sub-Saharan Africa (5). In addition, half of all low-birth-weight babies are born in South-central Asia, where 27 percent are below 2,500 g, while LBW levels in sub-Saharan Africa are estimated at 15 percent (6).

In East Africa, babies with low birth weight were found to account for 52% of newborn deaths. In Ethiopia in 2014, 17.1% of neonates were born with low birth weight. Low maternal education, Mothers having neonates from unmarried womens, and low household wealth were associated factors for neonatal mortality (7). In Ethiopia, neonatal mortality rates are higher than in other sub-Saharan African countries (8).

Ethiopia is the fourth highest neonatal mortality country in the world, and According to the 2019 mini EDHS report in Ethiopia, neonatal mortality was 33/per 1,000 live births (9).

In 2012, the world health assembly set a comprehensive implementation plan on maternal, infant, and young child nutrition, which specified six global nutrition targets, including a 30% reduction in LBW live births between 2012 and 2025 (10).

Low birth weight neonates are subjected to a greater risk of poor health, and death and require a longer period of hospitalization during their treatment (11). For many neonates, mainly low birth weight neonates, their day of birth is also their day of death with approximately 1 and 2 million deaths occurring on the day of their birth and in the first week of their life annually in the world respectively (12). The risk of death in the first 28 days of life was also seven times higher for babies born with low birth weight compared to those with normal birth weight (13).

There is considerable variation in the LBW neonatal survival rate across regions and within countries. Sub-Saharan Africa had the highest neonatal mortality rate in 2018 at 28 deaths per 1,000 live births, followed by Central and Southern Asia with 25 deaths per 1,000 live births (14). The survival of LBW neonates depends on a variety of factors, which might vary greatly with economic, socio-demographic, maternal medical obstetric, and clinical factors. Even though neonatal intensive care units increased in number significantly, LBW neonates were facing several deaths that could have been avoided by appropriate interventions on certain contributing factors (15).

The majority of research was done on the prevalence of low birth weight (LBW) and its associated factors, But little is known about the incidence and predictors of neonatal mortality among LBW neonates in the Amhara region. Therefore, this study aimed to estimate the incidence and predictors of mortality of low birth weight neonates admitted at North Shoa, Public Hospital in Ethiopia.

## Methods

### Study design, period, and setting

An institutional-based retrospective follow-up study was conducted from January 1, 2019, to December 30, 2021. The study was conducted at three hospitals of neonatal intensive care units in North Shoa Zone Amhara Region, Ethiopia. The selected hospitals are Debre Berhan Comprehensive, Mehal Meda General, and Enate General Hospital.

### Population

All low birth weight neonates that were admitted to the neonatal intensive care unit in a public hospital in North Shoa zone Amhara Region, Ethiopia were the source population.

Moreover, all low birth weight neonates admitted to the neonatal intensive care unit in selected hospitals found in the North Shoa Administration Zone from January 1/2019 to December 30/2021 were included.

### Inclusion and exclusion criteria

All low-birth-weight neonates with a gestational age greater than 28 weeks who were admitted to the neonatal intensive care unit (NICU) from January 1/2019 to December 30/2021 were included. However, Only incomplete chart records and records which were not available during the data collection period were excluded from this study.

### Sample size determination and sampling procedure

The sample size was determined using a double population proportion formula by using major predictor variables (home delivery, maternal age >35, sepsis and respiratory distress syndrome) from another study conducted at Bahirdar. Maternal age was considered an independent predictor of mortality since it gives a maximum sample size; from that study proportion of those exposed to Death was 38.7% and non-exposed to Death was 24.8% (15, 16) Using the Epi info software version 7, to one allocation ratio of exposed to non-exposed (1:1) was assumed. Finally, by using a 95% level of confidence, with a power of 80%, the total sample size was 416. A simple random sampling method was used to select three hospitals from 10 governmental hospitals, and LBW neonatal cards were proportionally allocated according to their total LBW neonatal admission at the NICU.

There were 2,507 neonates with low birth weight admitted to the NICU of three hospitals from 2019 to 2021. The neonate's medical record number (MRN) was used as a sampling frame. The sample was proportionally allocated for each hospital.

### Study variables

#### Dependent variables

Neonatal mortality among low birth weight

#### Independent variables

▻ Socio-demographic factors: Age of neonate, Sex, place of delivery, age of mother, residence.
▻ Maternal medical-related factors: Diabetes mellitus (DM), Hypertension, human immunodeficiency virus (HIV) Status of the mothers, and Anemia.
▻ Neonatal medical-related factors: Gestational Age, Congenital anomaly, neonatal Sepsis, Place of birth, Birth complication, perinatal Asphyxia, Respiratory Distress Syndrome (RDS), and Apgar score.
▻ Medical Obstetrics factors: Mode of delivery, Gravidity of mother, Parity, Premature rupture of membrane (PROM), prolonged labor, preeclampsia, maternal history of diabetic militus, human immunodeficiency virus (HIV), Antenatal care (ANC), and Twin Pregnancy.

### Operational definitions

Neonatal mortality: The death of the newborn from the time of birth up to 28 completed days of life.

Neonatal Respiratory Distress Syndrome: diagnosed based on the presence of one or more of the following signs: an abnormal respiratory rate, expiratory grunting, nasal flaring, chest wall recessions with or without cyanosis (17).

Event: Low birth weight neonates with the outcome of Death in the study period.

Censored: Low birth weight neonates with predictors other than Death (lost follow-up, survived over the follow-up period, referred to other health facilities against medical treatment).

Survival time: It is the time from admission to NICU with LBW to the occurrence of an event or censoring.

Survival status: Outcome of neonates, either death or censored.

Birth asphyxia: is diagnosed based on the World Health Organization (WHO) definition of “the inability to initiate and maintain breathing at birth” (18).

### Data collection tool and procedure

The data collection tool was taken from the standardized national neonatal and delivery room registration book. The checklist includes maternal and neonatal socio-demographic predictors, neonatal medical predictors, maternal medical and obstetric predictors, and the outcome of a low birth weight neonate, date of admission, and date of Death or censored. The first day of admission was used as the start of the follow-up period, while the last day of an event or censored occurrence was used as the end. All LBW neonatal medical registration numbers were obtained from the NICU ward. The required number of medical registration cards was chosen based on their eligibility criteria. The medical record office was then contacted to obtain selected medical cards. Finally, data collectors reviewed selected charts.

The data was gathered by three trained nurses who had Bachelor of Sciences in Nursing (BSCN) and were supervised by the principal investigator.

### Data quality control

A pretest with 5% of the sample population was conducted to ensure data quality. A 1-day training was given for data collectors and supervisors before the actual data collection. The supervisor carried out close supervision throughout the data collection time. The principal investigator entered and cleaned the data.

### Data processing and analysis

**Figure 1:**
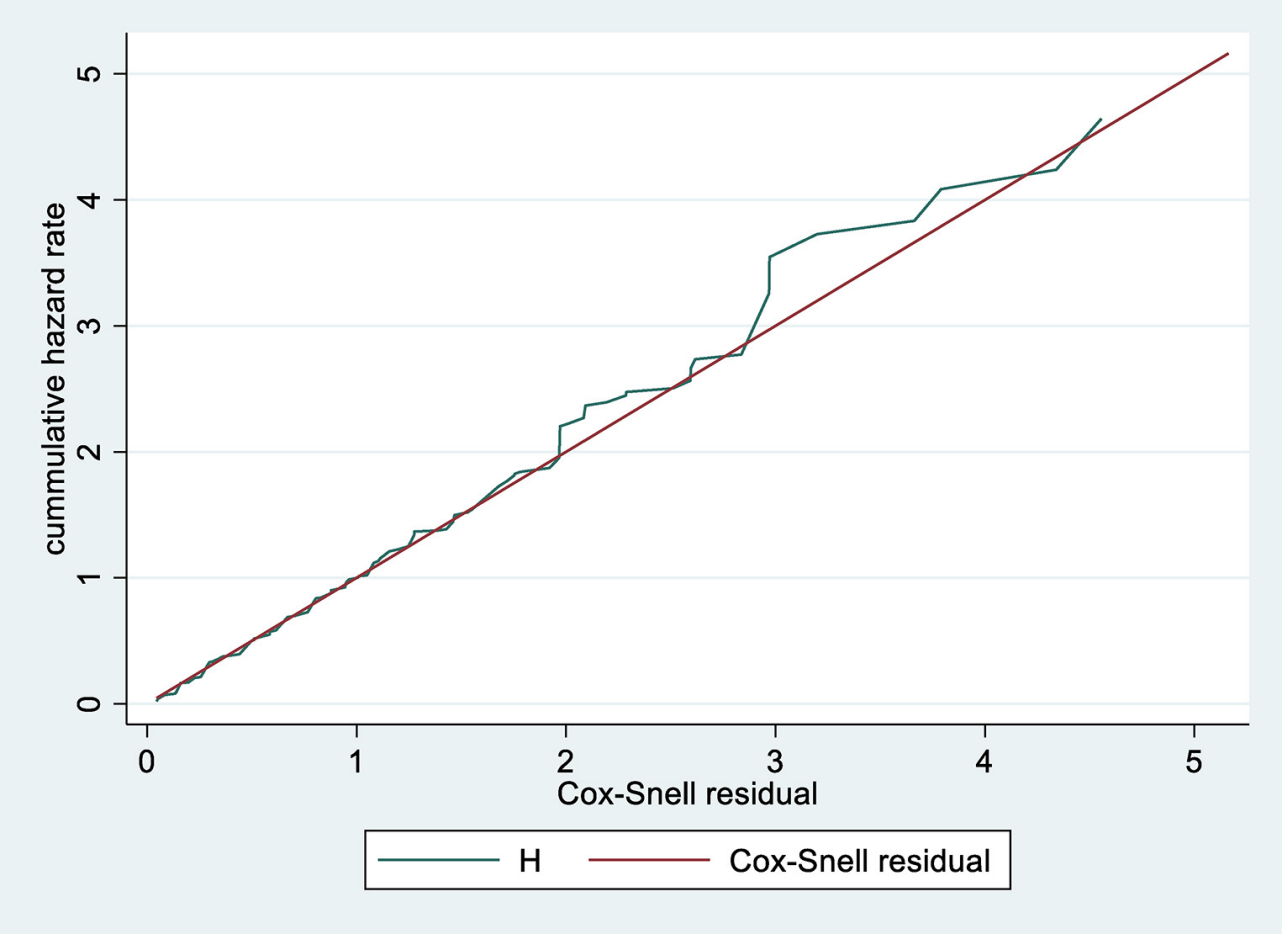
Cox-Snell residual cumulative hazard graph on neonates admitted in NICU at public North Shoa Hospital, from January 1st, 2019 to December 30, 2021.

Data were entered, cleaned, edited, and coded using Epi data version 4.2, Then exported to STATA 14 statistical software for analysis. Descriptive statistics (mean, standard deviation, median, frequency and percentages) were computed depending on the nature of the variable, and results were presented as graphs and tables. The outcome of each participant was dichotomized into censored or death. The incidence density rate (IDR) was calculated for the entire study period. Kaplan Meir plot with log-rank tests was used to compare survival curves. Before performing the Cox-proportional hazard regression, model goodness-of-fit was checked by Cox Snell residuals (Figure 1). Model fitness was tested by using Schoenfeld residuals test for proportional assumption and it was fitted with a total global test result of 0.4205.

Those variables with a *p*-value <0.05 were entered into the model. Multi-collinearity was also checked. For each independent predictor, Bi-variable Cox Proportional Hazard regression was performed. The variable with a *p*-value <0.25 in bivarable analysis was included in multivariable Cox proportional hazard regression. Adjusted hazard ratio with a 95% confidence interval and *p*-value <0.05 was used to measure the strength of association and identify statistical significance.

### Ethical approval

Ethical approval was obtained from the institutional review board of Asrat Woldeyes Health Science Campus (protocol number IRB-061). Informed consent was obtained from a legal guardian. This study was conducted per the Declaration of Helsinki. All methods were carried out per relevant guidelines and regulations.

## Results

### Socio-demographic characteristics of study subjects

During the follow-up time, 2,507 LBW neonates were admitted to selected hospitals. Out of these, 416 neonatal charts were reviewed. 324 (77.8%) were born from maternal Ages 18–35 years. The median age of mothers was 30 years, with an interquartile range of 9 years.

Among all mothers enrolled in the study, the majority of 301 (72.4%) mothers had given birth at a health institution. In addition, nearly half of the neonatal admissions occurred from 1 to 7 days of age (202, 48.56%) (Table 1).

**Table 1:** Socio-demographic characteristics of LBW neonates admitted to NICU at selected public hospitals in the North Shoa zone from 2019 to 2021 (*N* = 416).

Variable	Category	Frequency (%)	Survival status	
			Censored (%)	Death (%)
Sex	Male	229 (55.05%)	160 (51.7%)	69 (64.49%)
	Female	187 (44.95%)	149 (48.22%)	38 (35.51%)
Residence	Rural	253 (60.82%)	190 (61.49%)	63 (58.88%)
	Urban	163 (39.18%)	119 (38.5%)	44 (41.2%)
Neonatal age	<24 h	168 (40.38%)	117 (37.86%)	51 (47.66%)
	1–7 days	202 (48.56%)	153 (49.51%)	49 (45.79%)
	≥7 days	46 (11.06%)	39 (12.62%)	7 (6.54%)
Maternal age	<18	17 (4.1%)	14 (4.5%)	3 (2.8%)
	18–35	324 (77.8%)	238 (77.1%)	86 (80.3%)
	≥35	75 (18.2%)	57 (18.45%)	18 (16.8%)
Place of delivery	Health institution	302 (72.6%)	232 (75.4%)	70 (65.4%)
	Home	114 (27.4%)	77 (24.6%)	37 (34.5%)

Among 416 study participants, more than half (55.1%) of them were males. The majority of mothers, 324 (77.8%), were belonging to 18–35 years group. Among admitted neonates, 155 (37.26%) of them were in the gestational age (GA) group of very premature (28–32 weeks), of which 60 (56.07%) died, which was higher than premature (41.12%) and mature neonates (2.80%). The mean gestational age was 33.7 ± 2.5 SD weeks (95% CI: 33.53, 34.02) (Table 1).

### Maternal obstetrics characteristics

Among 416 mothers, 272 (65.38%) of them had ANC follow-ups at nearby health institutions. From the study, the majority of mothers who had ≥ two pregnancies accounted for 194 (46.63%) and among them, 67 (62.62%) died. Of all mothers included in the study, 228 (54.8%) of mothers were diagnosed with obstetric problems. Of all births, the highest percentage of birth was given by spontaneous vaginal delivery, 277 (66.59%) (Table 2).

**Table 2:** Obstetric characteristics of LBW neonates admitted to NICU at selected public hospitals in the North Shoa zone from 2019 to 2021 (*N* = 416).

Variable	Category	Frequency (%)	Survival status	
			Censored (%)	Death (%)
ANC	Yes	272 (65.38%)	213 (68.93%)	59 (55.14%)
	No	144 (34.62%)	96 (31.07%)	48 (44.86%)
Mode of delivery	SVD	277 (66.59%)	218 (70.55%)	59 (55.14%)
	Instrumental	54 (12.98%)	37 (11.97%)	17 (15.89%)
	C/S	85 (20.43%)	54 (17.48%)	31 (28.97%)
Twin pregnancy	Yes	194 (46.63%)	127 (41.10%)	67 (62.62%)
	No	222 (53.37%)	182 (58.90%)	40 (37.38%)
Gravidity	Prmigravida	80 (19.23%)	65 (21.04%)	15 (14.02%)
	Multigravida	336 (80.77%)	244 (78.96%)	92 (85.98)
Parity	Prmipara	94 (22.6%)	78 (25.57%)	16 (14.02%)
	Multipara	322 (77.4%)	231 (74.4%)	91 (85.98%)
Preeclampsia	Yes	143 (34.46%)	93 (30.10%)	50 (47.17%)
	No	272 (65.54%)	216 (69.9%)	56 (52.83%)
PROM	Yes	66 (15.87%)	44 (14.24%)	22 (20.56%)
	No	350 (84.13%)	265 (85.76%)	85 (79.44%)
Prolonged labour	Yes	88 (21.15%)	64 (20.71%)	24 (22.43%)
	NO	328 (78.85%)	245 (79.29%)	83 (77.57%)

SVD, spontaneous vaginal delivery; PROM, premature rapture of membrane.

### Maternal medical characteristics

Among the total mothers enrolled in this study, 107 (25.72%) mothers had HIV/AIDS, and 74 (17.79%) had Anemia, 105 (25.4%) of mothers had Diabetes mellitus (DM, and nearly half, 49.53%, of their neonates died. Furthermore, 108 (25.96%) mothers had a history of hypertension, and 77 (18.51%) mothers had a History of Tuberculosis (TB) (Table 3).

**Table 3:** Maternal medical diagnosis among neonates admitted to NICU at selected public hospitals in the North Shoa zone from 2019 to 2021 (*N* = 416).

Variable	Category	Frequency (%)	Survival status	
			Censored (%)	Death (%)
HIV	Yes	107 (25.72%)	58 (18.77%)	49 (45.79%)
	No	309 (74.28%)	251 (81.23%)	58 (54.21%)
Anemia	Yes	74 (17.79%)	46 (14.89%)	28 (26.17%)
	NO	342 (82.21%)	263 (85.11%)	79 (73.83%)
Tuberculosis	Yes	77 (18.51%)	50 (16.18%)	27 (25.23%)
	No	339 (81.49%)	259 (83.82%)	80 (74.77%)
Diabetes mellitus	Yes	109 (26.20%)	56 (18.12%)	53 (49.53%)
	No	307 (73.80%)	253 (81.88%)	54 (50.47%)
History of hypertension	Yes	108 (25.96%)	66 (21.36%)	42 (39.25%)
	No	308 (74.04%)	243 (78.64%)	65 (60.75%)

### Neonatal medical predictors

The significant admission medical problems documented among neonates admitted to NICU during the follow-up period were low Apgar scores (1st and 5th minute Apgar score <7, 186 (44.71%) and, 160 (38.46%), sepsis 204 (49.04%), PNA 136 (32.69%), Jaundice 80 (19.23% and RDS 141 (33.89%). Among admitted neonates with RDS, more than half, 54 (50.47%), died, and Sepsis was the leading cause of Death which was 68 (63.55%). The median neonatal birth weight at admission was 1,600 g, with an IQR of 500 g (Table 4).

**Table 4:** Neonatal medical and other characteristics of LBW neonates admitted to NICU at selected public hospitals in the north shoa zone from 2019 to 2021 (*N* = 416).

Variable	Category	Frequency (%)	Survival status	
			Censored (%)	Death (%)
Sepsis	Yes	204 (49.04%)	136 (44.01%)	68 (63.55%)
	No	212 (50.96%)	173 (55.99%)	39 (36.45%)
Jaundice	Yes	80 (19.23%)	53 (17.15%)	27 (25.23%)
	NO	336 (80.77%)	256 (82.85%)	80 (74.77%)
Respiratory distress syndrome	Yes	141 (33.89%)	87 (28.16%)	54 (50.47%)
	No	275 (66.11%)	222 (71.84%)	53 (49.53%)
Perinatal asphyxia	Yes	136 (32.69%)	102 (33.01%)	34 (31.78%)
	No	280 (67.310%)	207 (66.99%)	73 (68.22%)
Necrotizing enter colitis	Yes	126 (30.29%)	103 (33.33%)	23 (21.50%)
	No	290 (69.7%)	206 (66.67%)	84 (78.50%)
Hypothermia	Yes	165 (39.66%)	116 (37.54%)	49 (45.79%)
	No	251 (60.34%)	193 (62.46%)	58 (54.21%)

### Incidence of neonatal mortality

**Figure 2:**
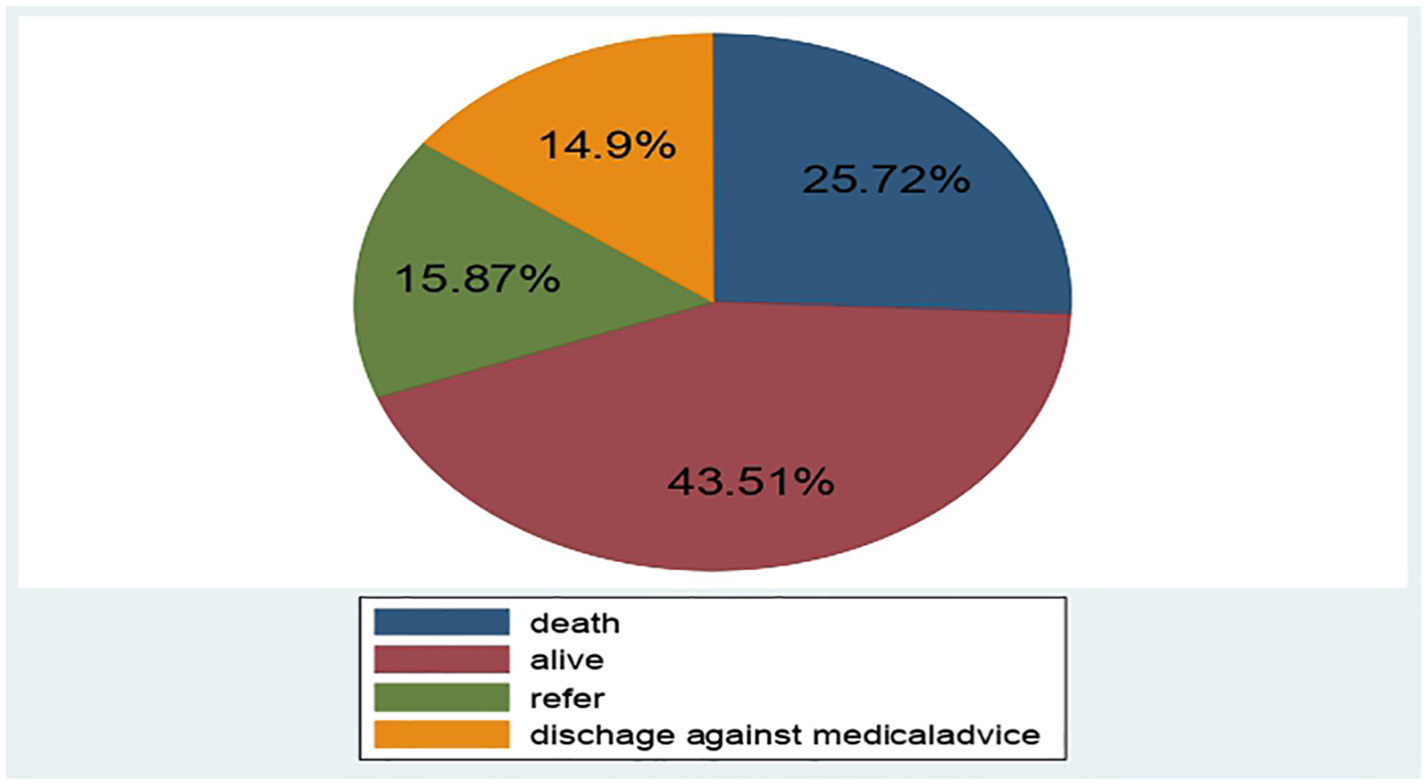
Outcome of LBW neonates admitted to NICU of public hospitals at North Shoa zone Hospital, Ethiopia, January 1st, 2019 to December 30, 2021.

A total of 416 LBW neonates were followed for 0–28 days. The overall proportion of neonatal mortality among the LBW neonate was 107 (25.72%) (95% CI: 21.50, 29.93) (Figure 2).

The overall incidence rate of mortality was 42.83 per 1,000 person-days (95% CI: 35.34, 51.77) with 2,498 person-days observation. In this study, the incidence rate of Mortality among LBW neonates within the first 24 h, 1–7 and 7–28 days were 60, 35 and 30 per 1,000 person-days observation, respectively (Table 5).

**Table 5:** Incidence density by birth weight and neonatal age among LBW neonates admitted to NICU at selected public hospitals in the north shoa zone from 2019 to 2021 (*N* = 416).

Variables	Category	Person-time in days	Incidence rate	Rate (95% CI) per 1,000
Birth weight	<1,000	261	36	137.93 (99.49, 191.21)
	1,000–1,499	1,232	58	47.71 (36.39, 60.89)
	1,500–2,499	1,005	13	12.93 (7.51, 22.27)
Neonatal age	≤24 h	841	51	60.64 (26.00, 45.28)
	1–7 days	1,424	49	35.11 (26.61, 46.32)
	≥7	233	7	30.04 (14.32, 63.01)
Gestational age				
28–32		888	60	67.5 (52.46, 87.02
33–36		1,469	44	29.99 (22.28, 40.24)
≥37		141	3	21.27 (6.86, 65.96)
Total incidence density		2,498	107	42.83 (35.34, 51.77)

The incidence rate of mortality among LBW neonates with 28–32 gestational age, 33–36 and ≥37 gestetional age were 60, 44 and 3 per 1,000 person-days observation respectively.

### Overall survival

**Figure 3:**
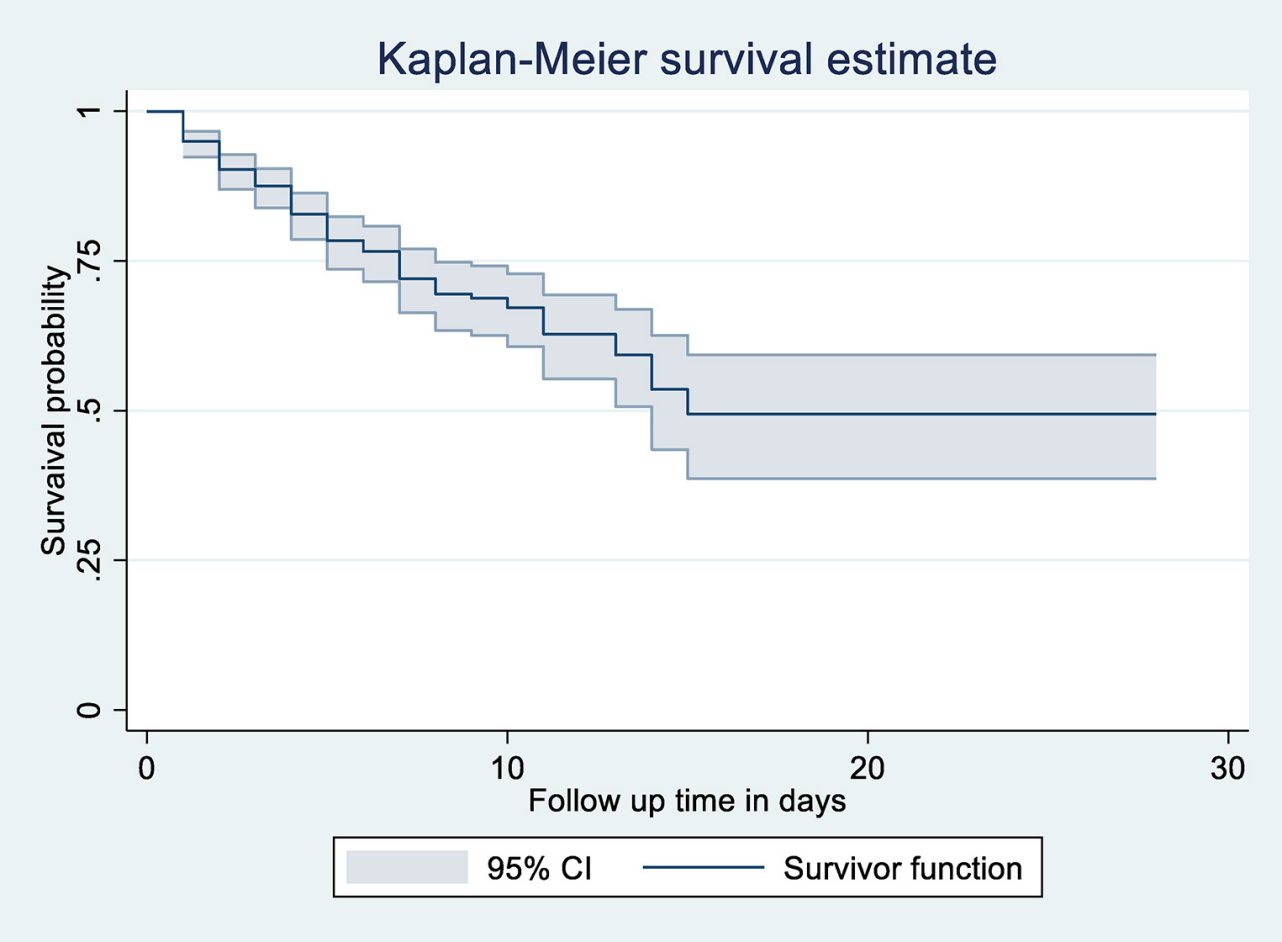
Overall Kaplan-Meier survival estimate of LBW neonates admitted to NICU at public Hospitals in North Shoa, Jannuary 1, 2019 to December 30, 2021.

The overall Kaplan–Meier estimate showed that LBW neonates’ survival probability was high on the first day of admission, which comparatively decreases as follow-up time increases (Figure 3).

During the first day of hospital stay, a maximum (94.95%) (95% CI: 92.36, 96.68) probability of survival was observed with a standard error of 0.01. The first quartile survival time was 7 days (95% CI: 5, 8). The overall survival ship probability of LBW neonates admitted to NICU throughout follow-up time remained at 49.43% (95% CI: 38.66, 59.31%).

The overall median survival time of neonates admitted to NICU in the study was 15 days (95% CI: 14) with a standard error of 1.37. This study also shows that the probability of neonatal survival on the 7th and 14th day of hospital stay was 72.06% (95% CI: 66.35, 76.98, SE = 0.02) and 53.3.03% (95% CI: 43.5, 62.5, SE = 0.049), respectively (Figure 2).

### Survival function among predictor variables

LBW neonates diagnosed with Respiratory Distress Syndrome (RDS) in this study had a mean survival time of 14 days (95% CI: 11).

At the 28 days of hospital stay, the overall survival of neonates with RDS and without RDS was 40.79% and 55.7%, respectively. This survival time difference was statistically significant with a *p*-value = 0.0012.

**Figure 4:**
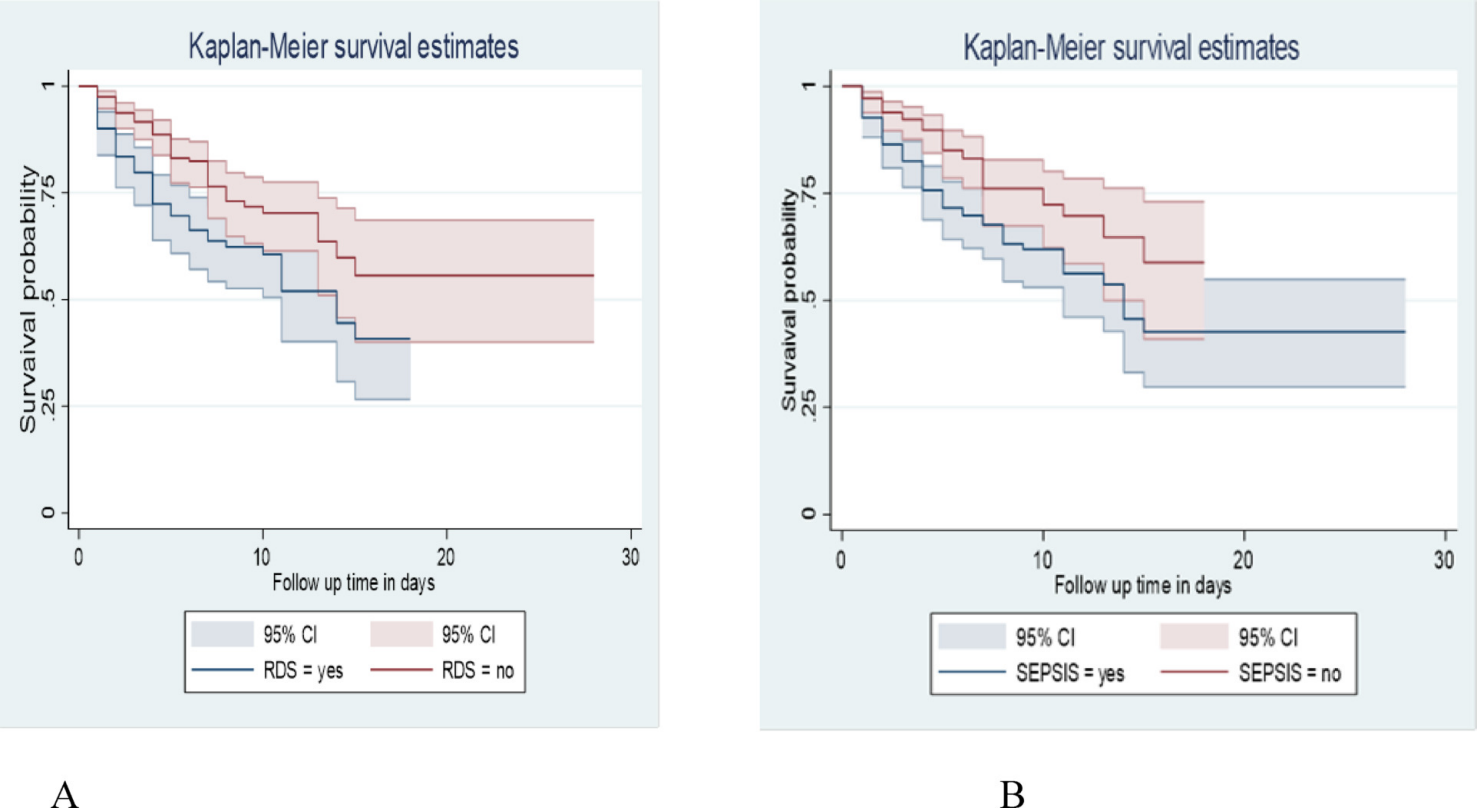
Kaplan-Meier survival estimate among LBW neonates admitted to NICU at public hospitals in the North Shoa zone with the category of **(A)**: RDS. **(B)**: sepsis from January 1, 2019 to December 30, 2021.

Neonates diagnosed with Sepsis had a lower survival time than those without Sepsis, with an overall survival status of 42.63% and 58.87%, respectively, at the end of the follow-up period. *P*-value = 0.0026 (Figure 4).

**Figure 5:**
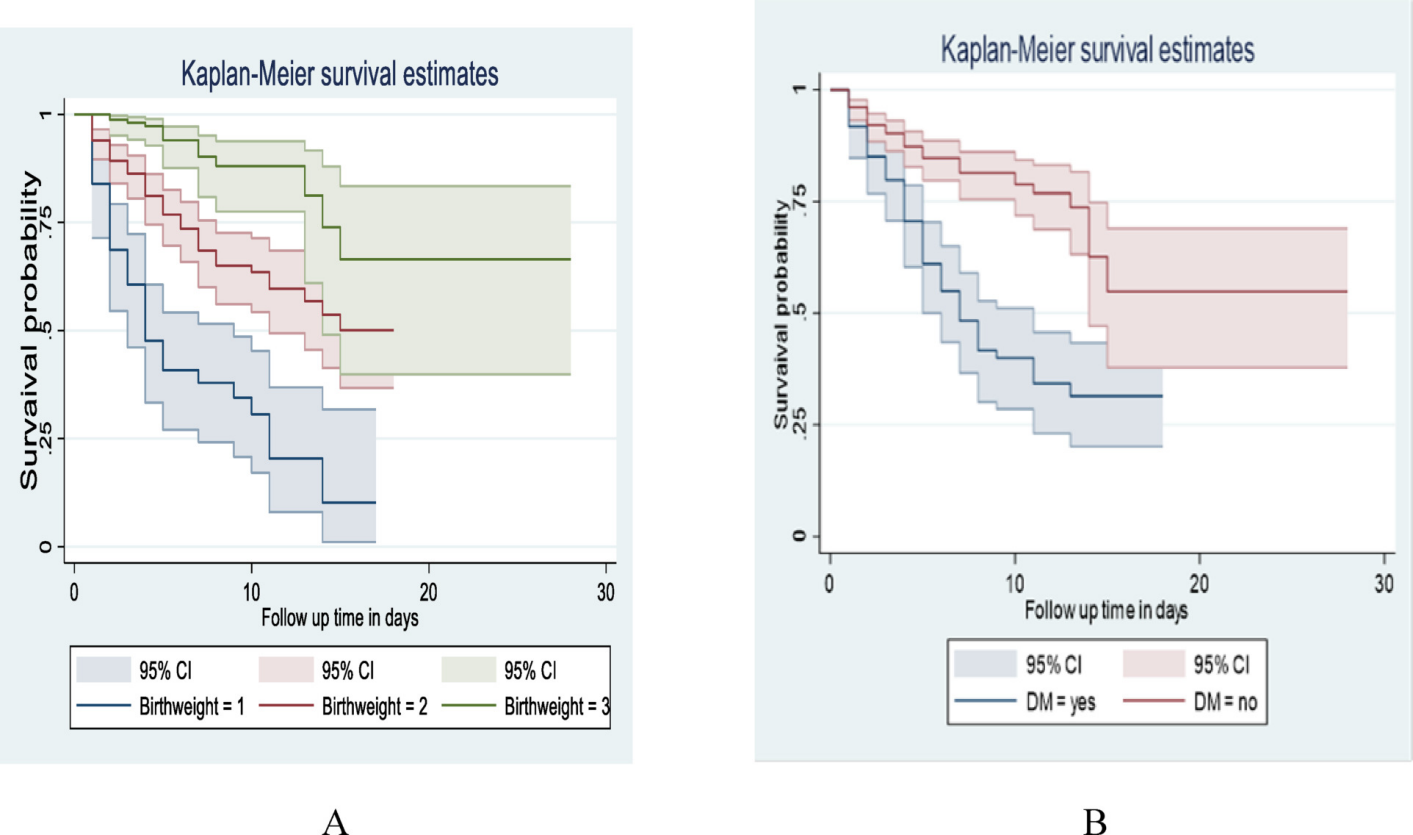
Kaplan-Meier survival estimate among LBW neonates admitted to NICU at public hospitals in North Shoa with the category of **(A)**: birth weight, **(B)**: maternal DM from January 1, 2019 to December 30, 2021.

This study also revealed that LBW neonates born from those mothers who hadn't Diabetes mellitus (DM) at baseline of admission had a longer survival time than those with DM (with a median of 7 days 95% CI: 5, 11) and (95% CI: 14) respectively. The Overall survival probability for LBW neonate mothers with DM and non-diabetic was 31.40% and 54.79%, respectively. This difference was statistically significant with a *p*-value = 0.000 (Figure 5).

### Cox proportional hazard model for different predictor variables

The Cox proportional hazard model was used to identify predictors of Mortality in LBW neonates. In bivariate Cox proportional hazard regression, sex, birth weight, neonatal age, place of delivery, Antenatal care follow-up, mode of delivery, parity, Gestational Age, multiple pregnancies, preeclampsia, maternal history of DM, maternal history of HIV, maternal history of hypertension, maternal history of Anemia, birth weight, Sepsis, jaundice, RDS and NEC were significant predictors of Mortality in LBW neonates at (*p*-value <0.25). In a multivariate proportional hazard model, neonatal sepsis, RDS, maternal history of DM, maternal history of HIV/AIDS, Twin pregnancy, preeclampsia, and extremely low birth weight, and Very low birth weight were independent predictors of LBW neonatal mortality at (*p* value <0.05).

Multivariate analysis revealed that neonates born with a birth weight less than 1,000 g were 8.4 times higher hazard of Death as compared to neonates weighing between 1,500 and 2,499 g (AHR: 8.4; 95% CI: 4.38, 16.14). LBW neonates born with birth weight between 1,000 and 1,499 g were 3.5 times higher hazard of Death than as compared to neonates weighing between 1,500 and 2,500 g (AHR: 3.5; 95% CI: 1.95, 6.51).

The hazard of Death in LBW neonates with Sepsis was 1.5 times higher than compared to those neonates without Sepsis (AHR: 1.53; 95% CI: 1.01, 2.32). Low birth weight neonates with RDS were also 1.6 times more at risk of dying than those without RDS (AHR: 1.6; 95% CI: 1.15, 2.63).

LBW neonates, Mothers with DM, were 2.5 times more hazardous to die than LBW neonate mothers without DM (AHR: 2.5; 95% CI: 1.70, 3.87). Low birth weight neonates with mothers with multiple pregnancies were 1.7 (AHR: 1.7; 95% CI: 1.42, 3.29) times riskier to die than those without multiple pregnancies. LBW neonates from Mothers with preeclampsia were 1.8 times more hazardous to die than mothers without preeclampsia (AHR: 1.83; 95% CI: 1.23, 2.73).

LBW neonates from Mothers with HIV were 2.2 times more hazardous to die than mothers without HIV (AHR: 2.2; 95% CI: 154, 3.42) (Table 6).

**Table 6:** Bivariate and multivariate cox proportional hazard regression of LBW admitted to NICU at selected public hospitals in the north shoa zone from 2019 to 2021 (*N* = 416).

Variable	Category	Status		CHR (95% CI)	AHR (95% CI)
		Censored	Death		
Sex	Male	160	69	1.3 (0.89, 2.01)*	1.4 (0.941, 2.16)
	Female	149	38	1	1
Place of delivery	Health institution	232	70	1	1
	Home	77	37	1.5 (1.03, 2.30)*	1.4 (0.84, 198)
Sepsis	Yes	136	68	1.8 (1.20, 2.71)*	1.5 (1.01, 2.32)**
	No	173	39	1	
RDS	Yes	87	54	1.8 (1.25, 2.681)*	1.6 (1.15, 2.63)**
	No	222	53	1	
NEC	Yes	103	23	0.7 (.43, 1.10)	0.68 (0.41, 1.13)
	No	206	84	1	
Maternal DM	Yes	56	53	2.8 (1.92, 4.12)	2.5 (1.70, 3.87)**
	No	253	54	1	
Maternal Anemia	Yes	46	28	1.5 (0.94, 2.30)*	1.4 (0.86, 2.17)
	No	263	79	1	
Twin pregnancy	Yes	127	67	1.9 (1.31, 2.88)*	1.7 (1.42, 3.29)**
	No	182	40	1	
Preeclampsia	Yes	93	50	1.7 (1.15, 2.47)	1.8 (1.23, 2.73)**
	No	216	56	1	
Maternal HIV	Yes	58	49	2.4 (1.64, 3.52)*	2.2 (1.54, 3.42)**
	No	251	58	1	

DM, diabetes mellitus; RDS, respiratory distress syndrome; NEC, necrotizing enterocolitis; HIV, human immunodeficiency virus.

N.B=:*Significant at (*p*-value <0.25), **Significant at (*p* value <0.05), 1, reference group.

## Discussion

This retrospective follow-up study aimed to assess incidence and predictors of neonatal mortality among LBW neonates admitted to the NICU of North Shoa zone public Hospitals.

The current study revealed that the overall incidence rate of LBW neonates was 42.83 deaths per thousand people per day observation (95% CI = 35.34, 51.77). The finding was in line with a study conducted in Bahirdar 35.4 per 1,000-person day observation (16). This similarity may be due to comparable socioeconomic conditions, healthcare access, and neonatal care practices within these Ethiopian settings. However, higher than the study conducted in Burkina Faso, 1.93 per thousand person-days observation (19), and india 21.22 per 1,000 (20). This variation may be due to study setting differences, quality of service among NICUs, study population differences and socio-demographic characteristics differences.

In this study, the highest proportion of death was recorded within <24 h of neonatal age (47.66%) (95% CI: 48.47, 62.21), which is consistent with the study conducted in India (51%). However, the proportion of Death within 1–7 days of age (45.79%) was higher than in the study conducted in India (13%) (21). The possible justification might be due to study population differences, study design and period.

This study identified several predictors of mortality. In this study hazard of Death among LBW neonates was enhanced by one point five due to the presence of Sepsis, which was similar to studies at Bahirdar (16), Northern India (21, 22). The possible justification might be that LBW neonates have decreased immune function because of the low level of some immune cells, such as immunoglobulin G resulting from the low transfer of maternal immunoglobulin G (23).

Low birth weight neonates with RDS at admission were one point six times the hazard of Death compared to LB neonates without RDS. This finding was supported by studies in Zimbabwe (24), Brazil (25), Iran, Indian studies, and Bahirdar (16, 21, 26). Furthermore, since most LBW neonates are preterm, the problem of lung immaturity is a common phenomenon that leads to lung collapse and respiratory failure (6).

Newborns with low birth weight who were been born by mothers with a history of diabetes mellitus have two points five times the higher hazard of mortality compared with the counterparts who did not have a history of diabetes mellitus (AHR: 2.5; 95% CI: 1.70, 3.87). This study finding is consistent with the study conducted at a public hospital in southern Ethiopia (27, 28). This might be related to the complications of diabetes mellitus during pregnancy because diabetes mellitus can result in hypoglycemia, hypocalcemia, respiratory distress, growth restriction, polycythemia, increased magnesium amount congenital anomalies, and excessively increased amount of bilirubin (29, 30).

LBW neonates from mothers with a history of HIV have two points two times the higher hazard of Death than their counterparts who did not have HIV (AHR: 2.2; 95% CI: 1.54, 3.42). This is consistent with the study conducted at Southern Ethiopia, the University of Gonder specialized hospital (AHR: 6.47; 95% CI: 1.43, 29.34) (31). This might be due to maternal immune compromisation, which limited the mother from breastfeeding and increased the burden of medical costs because newborns with low birth weight delivered to HIV-infected mothers can have 75% more cost than those newborns with low birth weight delivered from HIV-free mothers (32).

LBW neonates born from a mother with preeclampsia were nearly two times more likely to die than neonates whose mothers did not have preeclampsia. This result was consistent with a study done in Greece (33) and China (34). This is due to a decrease in uteroplacental blood flow, and the neonates develop hypoxia, and Intra-Uterine Growth Restriction (IUGR), which could increase the risk of dying (35).

In the current study, neonatal mortality hazard was higher among twin births than singletons. It was in line with a study reported in Ghana (36). This might be due to the increased risk of twins to prematurity, low birth weight, IUGR, twin-to-twin transfusion syndrome, and congenital anomaly that increases the risk of dying during the neonatal period than singletons (37) Besides, twins are more prone to malnutrition compared to singletons. Thus they are prone to infectious diseases, hypothermia, and Sepsis (38) which could increase their mortality risk during the neonatal period.

## Limitations of the study

The study was conducted using a secondary data source, important variables such as maternal educational status, maternal nutrition, cigarette smoking and chat chewing, and family income were missed due to chart incompleteness. In addition, this study was conducted in hospitals; neonates in the community were omitted, which may underestimate the mortality. Finally, we recommend that future researchers to conduct prospective studies and include facility-level treatment variations as a covariate.

## Conclusion

In this study, the LBW neonatal mortality incidence rate was high and continues as a public health issue. The significant predictors were sepsis, RDS, maternal history of DM, maternal history of HIV, preeclampsia and multiple pregnancies. Therefore, special attention should be given to those identified predictors of mortality, and different measures should be implemented to prevent premature birth in the high-risk population by improving prenatal care. Additionally, the Ministry of Health should focus on enhancing neonatal care by providing targeted training on neonatal protocols, particularly in the diagnosis and management of sepsis and respiratory distress syndrome (RDS).

## Data Availability

The raw data supporting the conclusions of this article will be made available by the authors, without undue reservation.
